# Laser-assisted see-through technology for locating sound sources inside a structure

**DOI:** 10.1038/s41598-024-53667-z

**Published:** 2024-02-16

**Authors:** Sean F. Wu, Yazhong Lu, Cameron Ernest, Yang Zhao, Lingguang Chen

**Affiliations:** 1 Department of Mechanical Engineering, Wayne State University, Detroit, MI 48202 USA; 2 School of Mechanical Engineering, Zhejiang University, Hangzhou, 310058 Zhejiang China; 3 Department of Electrical and Computer Engineering, Wayne State University, Detroit, MI 48202 USA; 4 Signal-Wise, LLC, Troy, MI 48098 USA

**Keywords:** Applied physics, Acoustics, Physics

## Abstract

A laser-assisted see-through technology is developed to locate sound sources inside a structure and to analyze the interior sound field. Six lasers were employed to measure simultaneously the normal velocities on the exterior surface. These input data were used to locate sound sources inside a solid structure using a passive sonic detection and ranging algorithm, and then to reconstruct the interior sound field using the Helmholtz equation least squares method, and finally to observe the changes of the interior sound field over time through computer tomography. If signals are time invariant, all these can be accomplished with two lasers, one being fixed and another moving around to measure the normal surface velocity sequentially to establish transfer function with respect to the stationary laser. Once the transfer functions are established, they can be multiplied by any segment of time-domain signals measured by the fixed laser to acquire multiple normal surface velocities, as if they were measured simultaneously. This laser-assisted see-through technology has been validated experimentally and employed to observe the aerodynamically-induced sound field generated by a blower inside a projector. This development is important as it signifies a significant advancement in sound source localization, and opens the door to a class of applications presently unattainable.

## Introduction

### Background

All state-of-the-art source localization methodologies, both active and passive, require that the line of sight between a sound source and any sensor be unblocked in a homogenous and isotropic elastic medium. If the line of sight is blocked, source localization cannot be done. Such a requirement has necessarily limited source localization to an obstacle-free space, regardless if it is an active source localization equipment such as radar^1–3^ to detect an aircraft in air and sonar to locate a submarine underwater^4–6^, or passive approaches, for example, triangulation-^7–9^ beamforming-^10–12^, time reversal-^13–15^, deep neural networks-based source localization methodologies^16–18^, including using robots to track and trace sound sources moving in 3D space^19–21^.

Customarily, there are two types of source localization methodologies: (1) by estimating TOA (time of arrival)^1–6,22^ of the signals measured by sensors; and (2) by estimating TDOA (time difference of arrival)^7–21,23^ of the signals measured by individual sensors. Active source localization utilizes TOA estimation, whereas passive source localization employs TDOA estimation.

Note that in all previous applications using TOA or TDOA, source localizations such as determining the impact location of a projectile on a plane^24–26^, the impact point on a plate^27–29^, detecting defects inside materials^30–32^, etc. were conducted within the same elastic medium. In other words, there are no impedance discontinuities in the elastic media.

In reality, however, sound sources are often inside solid structures, so the line of sight is blocked and there are impedance discontinuities between sound sources and sensors. For example, the root causes of noise and vibration issues of an engine block, a transaxle, a gearbox, etc. are inside solid structures. As such, source localizations cannot be done by using any existing methodologies. This is because the line of sight is blocked, and the presence of impedance discontinuities in the elastic media can have a negative impact on the convergence and accuracy of source localization results. Errors in source localizations may come from the refraction, reflection, and diffraction phenomena that force sound waves to travel in an unexpected direction. Also, different elastic media can have different types of elastic waves that travel at different speeds for different frequencies. All these phenomena will make it an extremely challenging task to model and predict the precise locations of sound sources. This is why source localization has traditionally been restricted to a homogeneous and isotropic medium, and the line of sight cannot be blocked.

Notice that in underwater applications, source localization can still be made when the line of sight is blocked by sea-mounts^33–35^ or even an island^36^, when certain conditions are met, for example, “*the diffracted energy of the signals from the island shadow would arrive at azimuths greater than the line of sight* (*LOS*) *bearing, whereas the refracted energy would arrive at azimuths smaller than the LOS bearing when the Lubell transducer moved out of the shadow*”^36^. In these cases, the source and receive are actually linked together by the same elastic medium, rather than completely blocked by an entirely different elastic medium.

### Theme

The major theme of this paper is to present a breakthrough discovery^37,38^ that enables one, for the first time ever, to locate sound sources and see sound fields inside solid structures. Such a discovery is unprecedented because not only is the line of sight completely blocked, but there are impedance discontinuities in the elastic media that completely separate sources and receivers.

Specifically, we employed six lasers to measure simultaneously the normal velocity on the surface of a structure, used passive SODAR (sonic detection and ranging) algorithms^39^ to locate sound sources inside the structure, and applied the Helmholtz equation least squares (HELS) method to reconstruct the interior sound field^40^, and CT (computer tomography) algorithm to scan both space and time simultaneously to watch how the sound field inside the structure changed over time. When signals are time invariant, we can accomplish these by using two lasers, one being fixed and another moving around to measure the normal surface velocity sequentially to establish transfer function with respect to the stationary laser. Once the transfer functions are established, they can be multiplied by any segment of time-domain signals measured by the fixed laser to acquire multiple normal surface velocities, as if they were measured simultaneously.

Unlike the standard triangulation algorithms, passive SODAR enables one to locate sound sources in the presence of random background noise^41,42^. This is because passive SODAR algorithms has incorporated special features such as denoising, redundancy checks, and optimizations to ensure optimal source localization under a non-ideal test condition. The hardware associated with passive SODAR includes six microphones in a 3D pentagon cone shape that enables one to not only acquire Cartesian coordinates of sound sources, but also reduce source localization errors by using the least-squares minimization^39^. Meanwhile, the HELS formulations always yield the optimal approximate reconstruction of the sound field for any given set of input data. The HELS formulations pursue an optimal approximation of the acoustic pressure by using an expansion of the spherical harmonics with reconstruction errors minimized by the least squares^40^. This is why it can be used to visualize the sound field emitted by an arbitrarily shaped vibrating structure under a non-idealized condition successfully and cost-effectively^43–45^, which cannot be done by using other methods such as the Fourier acoustics^46^ and boundary element method based nearfield acoustical holography^47^.

Note that there have been many studies that describe measurements of the sound pressure by using LDV (laser doppler vibrometer)^48–50^ rather than using a microphone; detection of the bearing angle of an incident acoustic pressure wave using LDV to interrogate an air–water interface^51–53^; measurement of vibration patterns of structures using LDV^54–56^; etc. These previous studies are different from what is presented here in that the former describes direct measurements of the sound pressure and vibration patterns of a structure using LDV, whereas the latter presents locating sound sources and reconstructing sound fields inside a completely enclosed structure, given the normal velocity measured by LDV on its exterior surface.

To the best of the authors’ knowledge, no such methodology and results have been reported in the literature to date.

### Significance

The significance of this study lies in the fact that it signifies an important breakthrough in the realm of sound source localization and sound field visualization. Invention of the see-through technology makes it possible for people to locate sound sources, visualize sound fields, and observe evolutions of the sound fields in 3D space over time inside a solid structure.

It is emphasized that this see-through technology enables one to quantify and rank source strengths in terms of SPL (sound pressure level) values, which is critically important in analyzing the root causes of noise and vibration issues inside complex machines. Such capabilities are made possible by combining passive SODAR, HELS methods, and CT scanning to not only locate sound sources, but also reconstruct the sound pressure field inside a solid structure. In contrast, all previous source localization methodologies, regardless of active or passive, can only provide the locations of sound sources but not their strengths in terms of SPL values.

We anticipate potential applications of this laser-assisted see-through technology to include but not limited to a class of vibroacoustic issues, such as determining the root causes of noise and vibration issues inside complex machines and structures, locating the partial discharges inside high voltage power transformer boxes, and many other non-contact, non-invasive diagnostic and product quality control tasks that cannot be accomplished easily by using traditional methodologies.

## Results

### Approaches

To lower the costs of experimental validations, we used six self-made laser vibrometers to measure the normal velocities on the surface of a vibrating structure simultaneously, and took them as input data to passive SODAR algorithms to locate sound sources inside the structure. The source locations were then taken as input to the HELS formulations to reconstruct the interior acoustic pressure field. Finally, we applied CT scan in both space and time domains simultaneously to observe the changes of the interior acoustic pressure field over time.

In what follows, we give a brief account of the approaches taken, including the major formulations used, to obtain the test results presented in this section.

#### Laser vibrometer

To prove the concept of this laser-assisted see-through technology, we need to take multiple normal surface velocities at different locations simultaneously. Although accelerometers can be an option, we preferred laser vibrometers as they were non-contact, non-invasive, and easy to control remotely. To reduce the costs of having multiple laser vibrometers, we used off-the-shelf items, designed, and assembled laser vibrometers ourselves.

**Figure 1:**
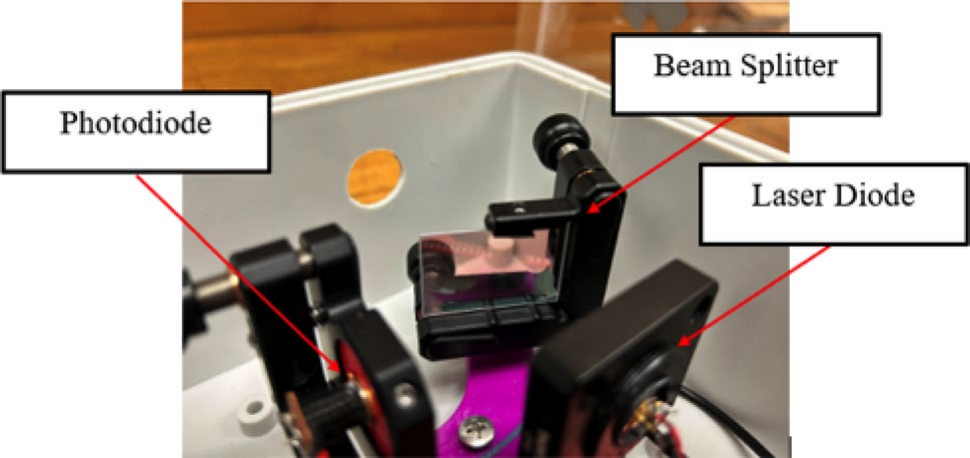
A home-made laser vibrometer module using off-the-shelf laser diode Model LTD505T, photodiode Model OP913SL, and a beam splitter. There were six laser vibrometer modules used in this study to measure the normal surface velocities on a solid structure simultaneously.

Figure 1 demonstrates the assembly of a laser vibrometer module, which includes laser diode Model LTD505T, photodiode Model OP913SL, and a beam splitter. The laser diode Model LTD505T is a single mode laser diode with a 5mW output power, 650 nm wavelength visible red beam, and 8 deg beam divergence after alignment.

#### Passive SODAR

Although passive SODAR algorithms contain special features to ensure optimal source localization under a non-ideal test condition, where there are random background noise and unknown interfering signals, the underlying principle is a triangulation to locate a sound source. In particular, a 3D array containing six microphones are used to collect input data, which leads to an over-determined system of equations. Meanwhile, the least squares minimization, redundancy check, windowing, filtering, and other signal processing techniques are used to minimize source localization errors to determine the Cartesian coordinates of multiple sound sources simultaneously in 3D space^39,41,42^,

At the core of passive SODAR algorithms is a triangulation formulation^39^,

1 $$\left| {\mathop{r}\limits^{\rightharpoonup} - \mathop{r{_j}}\limits^{\rightharpoonup}} \right| = \left| {\mathop{r}\limits^{\rightharpoonup} - \mathop{r_{i} }\limits^{\rightharpoonup} } \right| + c\Delta t_{i,j} , \,i,j = { }1,{ }2,{ } \ldots ,{ }L,\,{ }i \ne j,$$

where $\mathop{r}\limits^{\rightharpoonup}$, $\mathop{r _{i}}\limits^{\rightharpoonup}$, and $\mathop{r_{j}}\limits^{\rightharpoonup}$ are position vectors. For the *i*th and *j*th microphones under Cartesian coordinates they are expressible, respectively, as

2 $$\mathop{r}\limits^{\rightharpoonup} = x\mathop{e_{x}}\limits^{\rightharpoonup} + y\mathop{e_{y}}\limits^{\rightharpoonup} + z\mathop{e_{z}}\limits^{\rightharpoonup}, \;\; \mathop{r_{i}}\limits^{\rightharpoonup} = x_{i} \mathop{e_{x}}\limits^{\rightharpoonup} + y_{i} \mathop{e_{y}}\limits^{\rightharpoonup} + z_{i} \mathop{e_{z}}\limits^{\rightharpoonup},\;\; \mathop{r_{j}}\limits^{\rightharpoonup} = x_{j} \mathop{e_{x}}\limits^{\rightharpoonup} + y_{j} \mathop{e_{y}}\limits^{\rightharpoonup} + z_{j} \mathop{e_{z}}\limits^{\rightharpoonup}.$$

The quantity *c* $\Delta$ t_*i*,*j*_ in Eq. (1) represents the distances travelled by the sound waves from the *i*th to *j*th microphone, and *c* is the speed of sound in air at any given temperature. Equation (1) can be solved explicitly (see Appendix A of Ref.^39^) and the final results are given below for completeness.

3 $$\left(x,y,z\right)={\text{Average}}\left[\left({x}_{p},{y}_{p}, {z}_{p}\right),\left({x}_{q},{y}_{q}, {z}_{q}\right)\right],$$

where both (*x*_*p*_,* y*_*p*_,* z*_*p*_) and (*x*_*q*_,* y*_*q*_,* z*_*q*_) are candidates for the optimal solution to a target sound source in 3D space. In a free field under a high SNR (signal to noise ratio), these two solutions overlap each other. In practice, however, this is not the case because there are random background noise, interfering signals, sound reflections and reverberations from unspecified surfaces, which can lower SNR and lead to multiple solutions to the source location. Under these conditions, a spatial average is taken as the optimal solution to an approximate source location as shown in Eq. (3).

It is emphasized that Eq. (3) is valid for a very wide frequency range. Experimental validations have demonstrated that passive SODAR algorithms may be very effective in locating both airborne and structure-borne noise sources under non-ideal test conditions^57–59^. Moreover, it has been applied successfully to diagnose and analyzes complex automobile noise under constant accelerating^60^ and sudden accelerating conditions^61^.

#### The HELS method

The HELS method seeks the optimum reconstruction of the acoustic field emitted by arbitrary sound sources in non-ideal environment using an expansion of the spherical harmonics with reconstruction errors minimized by the least squares^40^. Mathematically, the HELS formulations are expressible as,

4 $$p\left(\mathbf{x};\omega \right)=\sum_{j=1}^{J}{\Psi }_{j}\left(\mathbf{x};\omega \right){C}_{j}\left(\omega \right), j = 1, 2, \dots , J,$$

where *ω* indicates the angular frequency, ${\Psi }_{j}\left(\mathbf{x};\omega \right)$ represents the *j*^th^ basis function that satisfies the Helmholtz equation at a field point **x** in space, and ${C}_{j}\left(\omega \right)$ depicts the *j*^th^ expansion coefficient, which are determined by taking measurements of the acoustic pressure $p\left({\mathbf{x}}_{m}^{meas};\omega \right)$ at ${\mathbf{x}}_{m}^{meas}$, *m* = 1, 2, …, *M*, and performing a pseudo inversion of Eq. (4). In a matrix form, {*C*(*ω*)}_*J*×1_ is expressible as,

5 $$\left\{ {C\left( \omega \right)} \right\}_{J \times 1} = \left[ {\Psi \left( {x_{m}^{meas} ;\omega } \right)} \right]_{J \times M}^{\dag } \left\{ {p\left( {x_{m}^{meas} ;\omega } \right)} \right\}_{M \times 1} ,$$

where {*C*(*ω*)}_*J*×1_ is a column vector for the expansion coefficients *C*_*j*_(*ω*), ${\left\{p\left({\mathbf{x}}_{m}^{meas};\omega \right)\right\}}_{M\times 1}$ depicts the column vector of the measured acoustic pressure at ${\mathbf{x}}_{m}^{meas}$, and $\left[ {{\Psi }\left( {{\mathbf{x}}_{m}^{meas} ;\omega } \right)} \right]_{J \times M}^{\dag }$ is the pseudo inversion matrix given by,

6 $$\left[ {\Psi \left( {x_{m}^{meas} ;\omega } \right)} \right]_{J \times M}^{\dag } = \left( {\left[ {\Psi \left( {x_{m}^{meas} ;\omega } \right)} \right]_{J \times M}^{H} \left[ {\Psi \left( {x_{m}^{meas} ;\omega } \right)} \right]_{M \times J} } \right)^{ - 1} \left[ {\Psi \left( {x_{m}^{meas} ;\omega } \right)} \right]_{J \times M}^{H} ,$$

where ${\left[\Psi \left({\mathbf{x}}_{m}^{meas};\omega \right)\right]}_{M\times J}$ is a rectangular matrix that contains the basis functions $\Psi \left({\mathbf{x}}_{m}^{meas};\omega \right)$ with a superscript *H* indicating a Hermitian transposition.

Substituting Eq. (5) into (4) yields the reconstructed acoustic pressure at any field point ${\mathbf{x}}_{n}^{rec}$, *n* = 1, 2, …, *N*.

7 $${\left\{p\left({\mathbf{x}}_{n}^{rec};\omega \right)\right\}}_{N\times 1}={\left[{G}_{pp}\left({\mathbf{x}}_{n}^{rec}\left|{\mathbf{x}}_{m}^{meas};\omega \right.\right)\right]}_{N\times M}{\left\{p\left({\mathbf{x}}_{m}^{meas};\omega \right)\right\}}_{M\times 1},$$

where ${\left[{G}_{pp}\left({\mathbf{x}}_{n}^{rec}\left|{\mathbf{x}}_{m}^{meas};\omega \right.\right)\right]}_{N\times M}$ is known as the pressure-to-pressure transfer function,

8 $$\left[ {G_{pp} \left( {x_{n}^{rec} \left| {x_{m}^{meas} ;\omega } \right.} \right)} \right]_{N \times M} = \left[ {\Psi \left( {x_{n}^{rec} ;\omega } \right)} \right]_{N \times J} \left[ {\Psi \left( {x_{m}^{meas} ;\omega } \right)} \right]_{J \times M}^{\dag } .$$

In practice, the measured acoustic pressures contain errors and the input data are always insufficient to completely define the acoustic pressure field. Therefore, reconstruction of the acoustic pressure, Eq. (4), is an ill-posed problem. To overcome this ill-posedness difficulty, regularization is utilized. There are many regularization schemes such as truncated singular value decomposition^62^, weighted singular value decomposition ^63^, Tikhonov regularization^64^, modified Tikhonov regularization^65^, etc. Here, we choose the least squares minimization to determine the optimal number of expansion terms to reconstruct the acoustic pressure because it is simple to implement,

9 $${\epsilon }_{p}=\underset{{J}_{{\text{op}},p}}{{\text{min}}}{\Vert \left\{{\left[\mathbf{I}\right]}_{M\times M}-{\left[{G}_{pp}\left({\mathbf{x}}_{m}^{rec}\left|{\mathbf{x}}_{m}^{meas};\omega \right.\right)\right]}_{M\times M}\right\}{\left\{p\left({\mathbf{x}}_{m}^{meas};\omega \right)\right\}}_{M\times 1}\Vert }^{2},$$

where ${\epsilon }_{p}$ denotes the minimum error in reconstructing the acoustic pressure $p\left({\mathbf{x}}_{n}^{rec};\omega \right)$ at ${\mathbf{x}}_{n}^{rec}$ by optimizing the number of expansion terms ${J}_{{\text{op}}, p}$ through the least squares method to reconstruct the acoustic pressure $p\left({\mathbf{x}}_{n}^{rec};\omega \right)$, ${\left[\mathbf{I}\right]}_{M\times M}$ depicts a unitary matrix, and ${\left[{G}_{pp}\left({\mathbf{x}}_{m}^{rec}\left|{\mathbf{x}}_{m}^{meas};\omega \right.\right)\right]}_{M\times M}$ is the transfer function that correlates the measured acoustic pressure $p\left({\mathbf{x}}_{m}^{meas};\omega \right)$ at ${\mathbf{x}}_{m}^{meas}$ to the reconstructed acoustic pressure $p\left({\mathbf{x}}_{n}^{rec};\omega \right)$ at ${\mathbf{x}}_{n}^{rec}$.

This HELS method has been analyzed theoretically^40^ and proven experimentally in reconstructing the acoustic pressure field inside an arbitrarily shaped automobile^43,66^.

### Test setup

**Figure 2:**
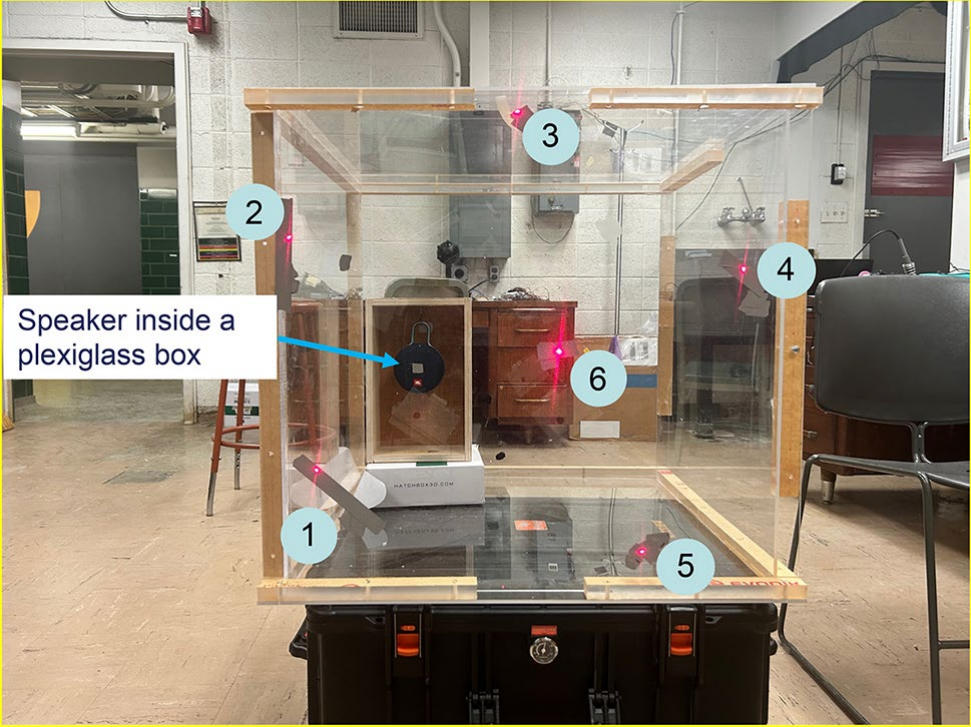
Test setup for locating the loudspeaker positioned at an arbitrarily selected place inside a completely sealed Plexiglas box. The positions of six measurement points were as indicated, whose coordinates were determined manually. The vibration signals measured by six lasers were taken as input to passive SODAR algorithms to locate the loudspeaker inside the Plexiglas box.

Figure 2 displays the test setup to validate this laser-assisted see-through technology to locate sound sources inside a tightly sealed box of 61 × 61 × 61 cm^3^ made of Plexiglas of a 1 cm in thickness. The reason for choosing Plexiglass is because it is opaque to sound, but transparent to light so as to facilitate examinations of the accuracy in source localization.

We used six laser diodes that emitted six collimated visible red laser beams to measure the normal velocity on the surface of the Plexiglas simultaneously at a distance of 25 cm to ensure an acceptable SNR. Measurement points were exhibited in Fig. 2 and their coordinates were determined manually. Vibrations of the Plexiglas surfaces were generated by the incident sound waves emitted from a loudspeaker positioned at an arbitrarily selected location (see Fig. 2). The signals emitted from the loudspeaker included narrowband, broadband, random, impulsive, continuous, stationary, and transient. Six external photodiodes were utilized to monitor the vibration signals measured by laser beams, which were then taken as input to passive SODAR algorithms to locate the loudspeaker position inside the Plexiglas box. Tests were conducted inside a laboratory in the presence of random background noise and unspecified interfering signals.

### Experimental validations

**Figure 3:**
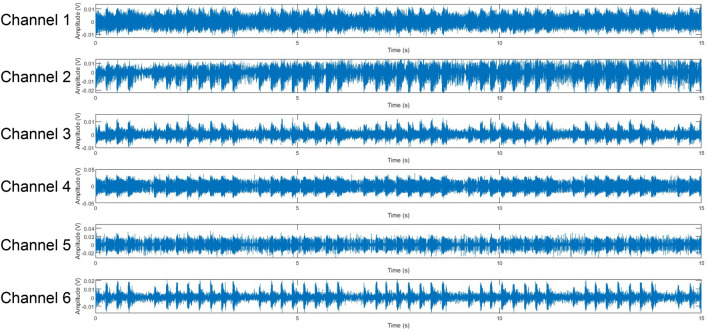
Examples of the signals measured by six laser vibrometers simultaneously.

**Figure 4:**
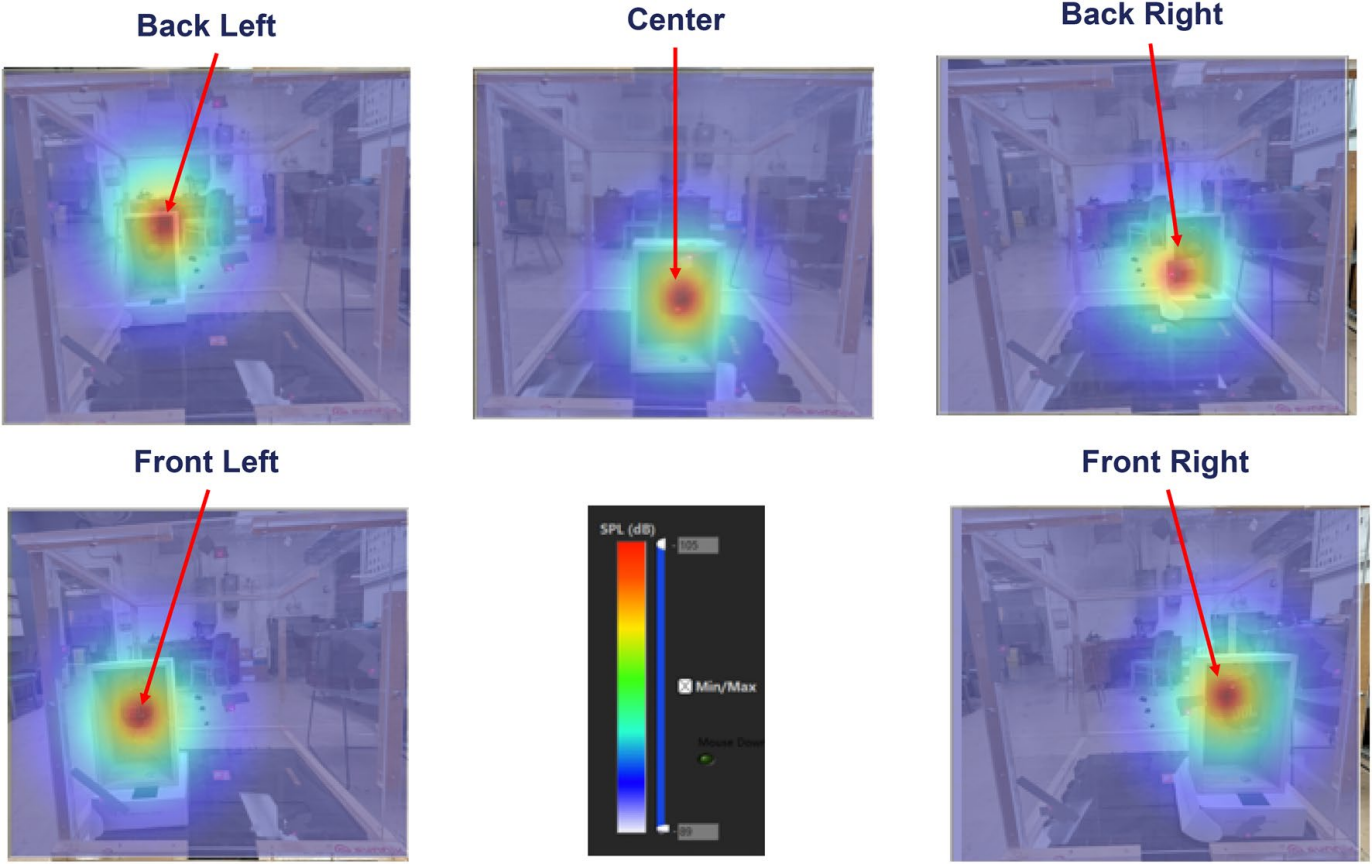
Results showed that no matter what signals were selected and where the loudspeaker was positioned inside this tightly sealed Plexiglas box, we could always pinpoint the precise location of this loudspeaker, regardless if it was at left, right, front, back, or center.

Figure 3 shows an example of the signals measured by six laser vibrometers simultaneously. These results demonstrate that SNR in each channel can be very different, depending on the measurement locations. For example, in the case as demonstrated in Fig. 3, SNR in channel 6 is much higher than the rest channels. However, no matter how differently the SNR values were, what signals were utilized, and where the loudspeaker was placed inside this sealed Plexiglas box, we could always pinpoint the location of the loudspeaker, regardless if it was at left, right, front, back, or center (see Fig. 4). Red color represented the highest amplitude of the acoustic pressure inside the Plexiglas box, which coincided with the loudspeaker position, and blue color depicted the lowest amplitude of the acoustic pressure. The color scale for SPL (Sound Pressure Level) values here ranged from 89 to 105 dB. More experimental validations and detailed descriptions of designs of experiments and measurement results can be found in References^67,68^.

Notice that quantification of the acoustic pressure field inside the Plexiglas box was done by using the HELS formulation, Eq. (4), based on the input data provided by six laser vibrometers. Once the source location is specified, the acoustic pressure field can be reconstructed by using an expansion of the basis functions that satisfy the Helmholtz equation, which for interior problems is expressible as the spherical Bessel functions and spherical harmonics using the spherical coordinates^40^,

10 $${\Psi }_{ij}\left(\mathbf{x};\omega \right)\equiv {\Psi }_{nl}\left(r,\theta ,\phi ;\omega \right)={j}_{n}^{\left(1\right)}\left(kr\right){Y}_{n}^{l}\left(r,\theta ,\phi \right),$$

where ${j}_{n}^{\left(1\right)}\left(kr\right)$ and ${Y}_{n}^{m}\left(r,\theta ,\phi \right)$ represent the spherical Bessel functions and spherical harmonics of the first kind, respectively, *n* and *l* indicate the indices, where *n* = 1, 2, …, *N*; and *l* ranges from $-n$ to *n*^40^. The optimal number of expansion ${J}_{{\text{op}}, p}$ is determined by Eq. (9) via the least squares minimization process.

It is emphasized that by using this see-through technology, we are not only able to locate the sound sources inside a sealed solid enclosure, but also quantify the interior acoustic pressure distribution. This is an important advancement over the existing source localization methodologies.

As a comparison, we ran these same tests by using six microphones, and took the acoustic pressure signals measured outside the Plexiglas box as input data to passive SODAR algorithms to locate the loudspeaker inside. However, we cannot determine the loudspeaker position correctly. This further reinforces the long-held belief that we cannot locate sound sources correctly when the line of sight between a source and receiver is blocked, or when there are impedance discontinuities in the elastic media between the source and sensors.

On the other hand, these experiments prove unambiguously that we can use the laser-assisted see-through technology to pinpoint sound sources even when the line of sight is blocked and there are impedance discontinuities in elastic media. The reason that the barriers in sound source localization can be broken is because measurements are taken on an impenetrable surface, on which the normal surface velocity is continuous across its thickness. As such, we can use the normal surface velocity measured on the front as that on the back side of the surface, and supply them to the passive SODAR algorithms to locate sound sources behind, thus bypassing this impenetrable surface.

This discovery is unprecedented, and its significance is that it opens the door to a class of potential applications that are currently unattainable.

### Seeing sound inside a projector

**Figure 5:**
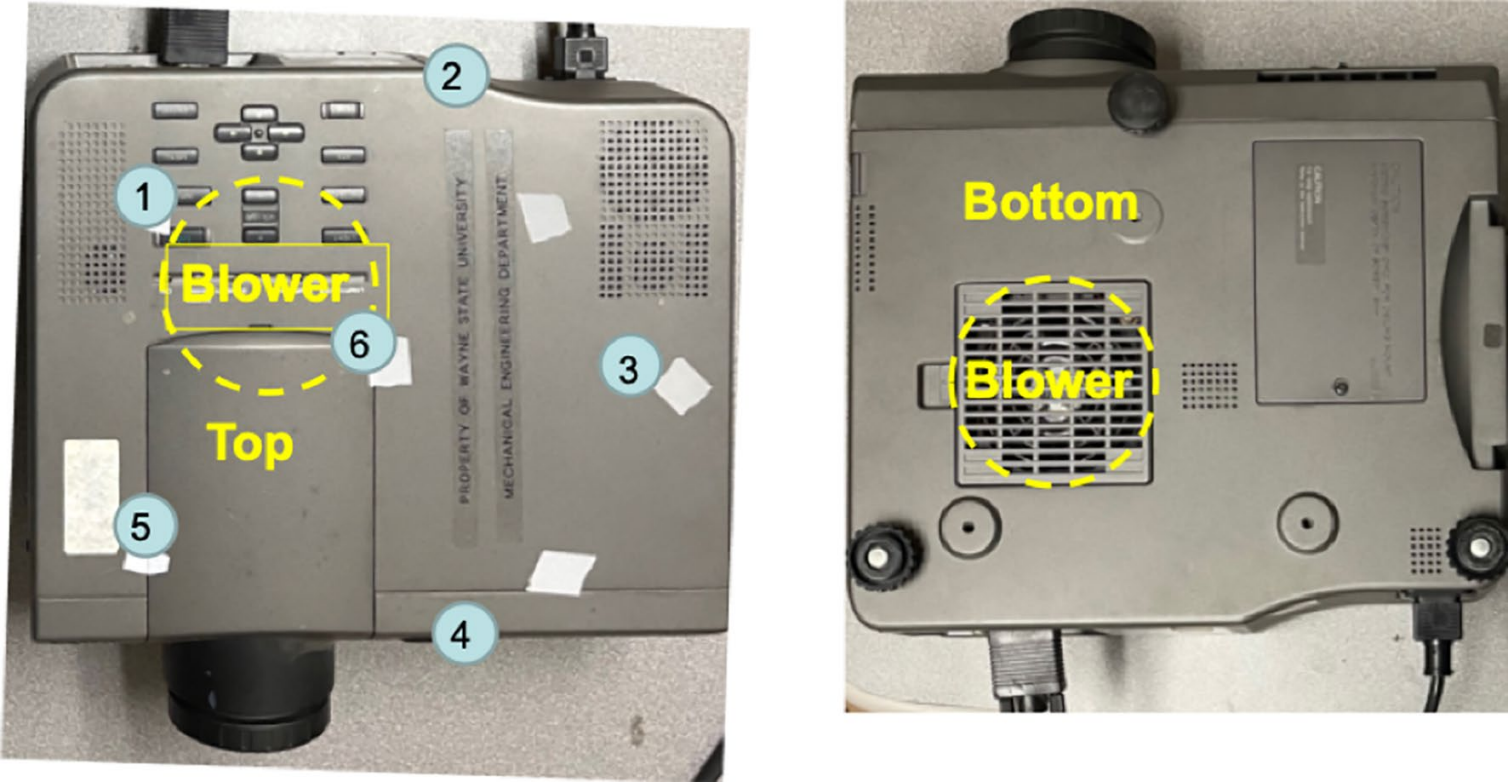
(**a**) The top view of the projector. (**b**) The bottom view of the projector. Measurement locations were given by numbers: 1, 3, 5, and 6 on the top surface; and 2 and 4 on the side (vertical) surfaces of the projector, respectively, and their coordinates were determined manually.

**Figure 6:**
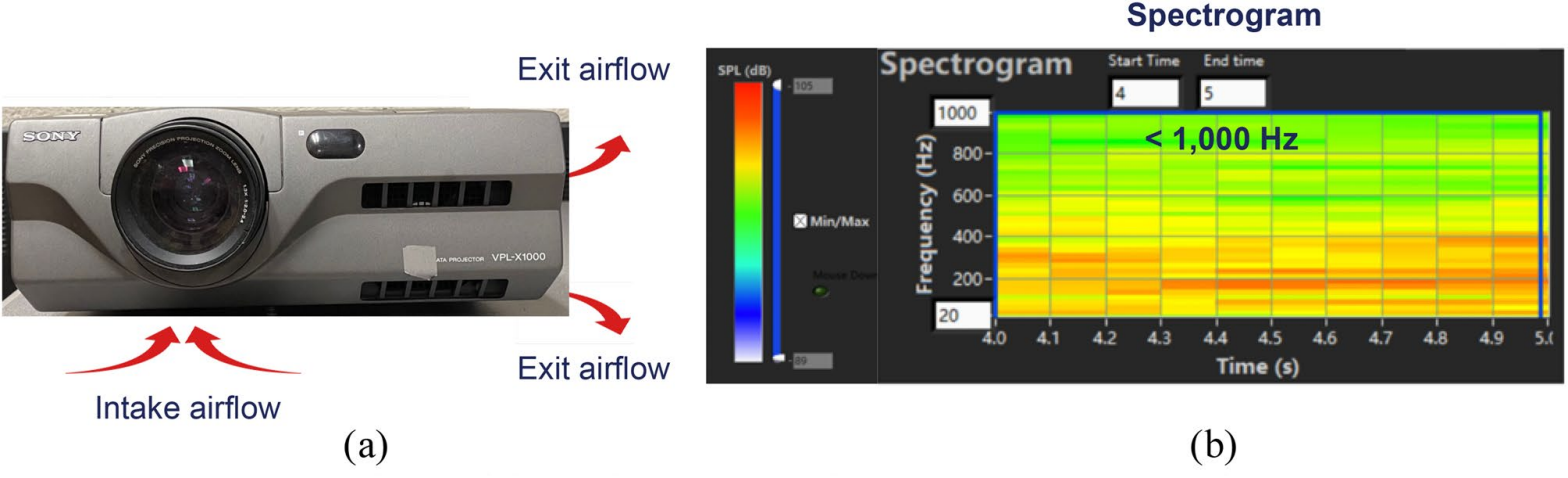
(**a**) Front view of the projector. Airflows were drawn into the projector from the bottom and ejected through front and top grids of the projector. (**b**) Spectrogram of the aerodynamically-generated sound produced by airflows inside the projector. Because blower speed was constant and low, so was the Reynold’s number. Thus, the frequencies of aerodynamically-generated sound were below 1000 Hz.

Next, we applied the laser-assisted see-through technology to see sound inside a standard projector. Figures 5a and 5b show the top and bottom sides of the projector, respectively. From Fig. 5b, we can see a grid, inside of which is a blower that draws airflows from the outside to lower temperature inside, and ejects them through outlets on the front and top surfaces, respectively. The entire unit is housed inside a metal cover. As the blower blades rotate, airflows are generated inside the projector, creating aerodynamically-generated sounds. Since the blower speed is constant and very low, so is the Reynold’s number. Accordingly, the frequencies of the aerodynamically-generated sound are below 1000 Hz (see Fig. 6b), where the spectrogram was obtained by using the standard short-time Fourier transform^69^.

To see sound inside the projector, we took six measurements of the normal velocities on the exterior surfaces of the projector. Measurement locations were shown by numbers: 1, 3, 5, and 6 on the top surface; and 2 and 4 on the side (vertical) surfaces of the projector, respectively (see Fig. 5). The coordinates of these measurement points were determined manually.

These measured time-domain vibration signals were used as input to passive SODAR algorithms to locate sound sources, which were taken as input to the HELS formulations to reconstruct the interior sound field. Because blower speed was constant and low, so was the Reynold’s number. Hence, the frequencies of aerodynamically-generated sound were below 1000 Hz.

Because the blower speed was constant and relatively low, signals were more or less time invariant. Under this condition, two laser vibrometers would suffice to measure the normal surface velocities. For example, one measurement point can be fixed at position #6 and another can take measurements at points #1, #2, #3, #4, and #5 sequentially to establish transfer functions at these locations with respect to the normal surface velocity measured at position #6. Multiplying these transfer functions by any segment of time-domain signals measured at position #6, we can obtain time-domain signals at points #1, #2, #3, #4, and #5, respectively, and achieve the same effects as those of using six laser vibrometers simultaneously.

**Figure 7:**
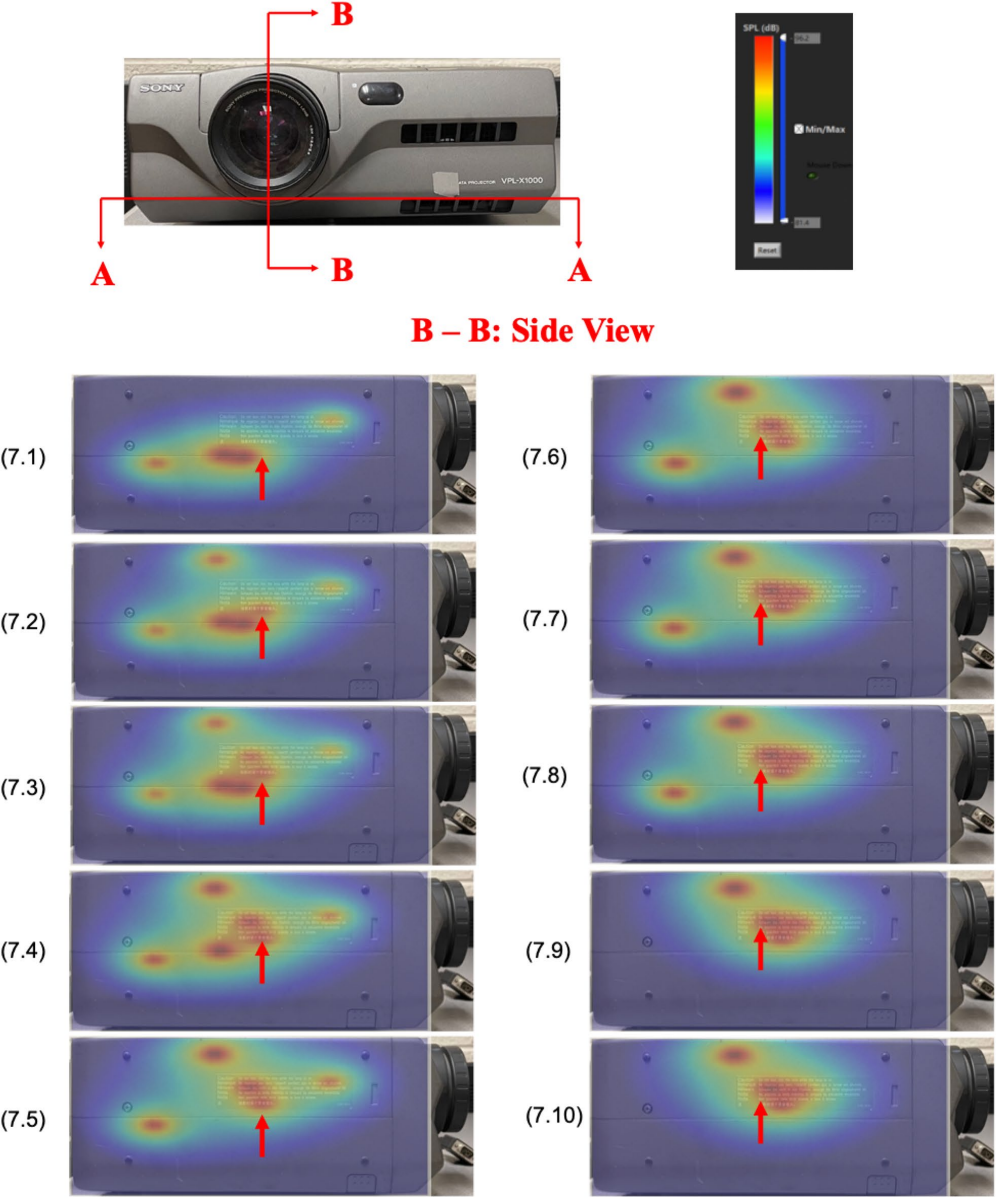
Evolution of reconstructed acoustic pressure distributions on B-B cross section over 1 s time with a constant interval of 0.1 s. As the blower blades drew airflows from the bottom, the aerodynamically-generated sound was seen to immerge from the plane of blower blades, move upwards, and dissipate towards the top as shown by dashed-red arrows. This motion was cyclic in time. The color scale depicted SPL values ranging from 81.4 to 96.2 dB.

To see how the interior sound field changed in space and time, we used CT scans in both space and time domains simultaneously. Figure 7 demonstrates two scanning cross sections, one being parallel (A-A) and another being perpendicular (B-B) to the plane of blower blades. On each of these cross sections, we scanned time at an interval of 0.1 s, which was sufficient to reveal detailed changes in the aerodynamically-generated sound field inside the projector. The entire time segment was 1 s.

Figure 7 demonstrates how the aerodynamically-generated sound field was changing on B-B cross section over time. The color scale depicted SPL values ranging from 81.4 to 96.2 dB. Red arrows indicated that the acoustic pressures emerged from blower blades, drifted upwards, and diminished toward the top. This motion appeared to be cyclic. The entire time segment was arbitrarily selected to show the evolution of the interior sound field inside a projector.

**Figure 8:**
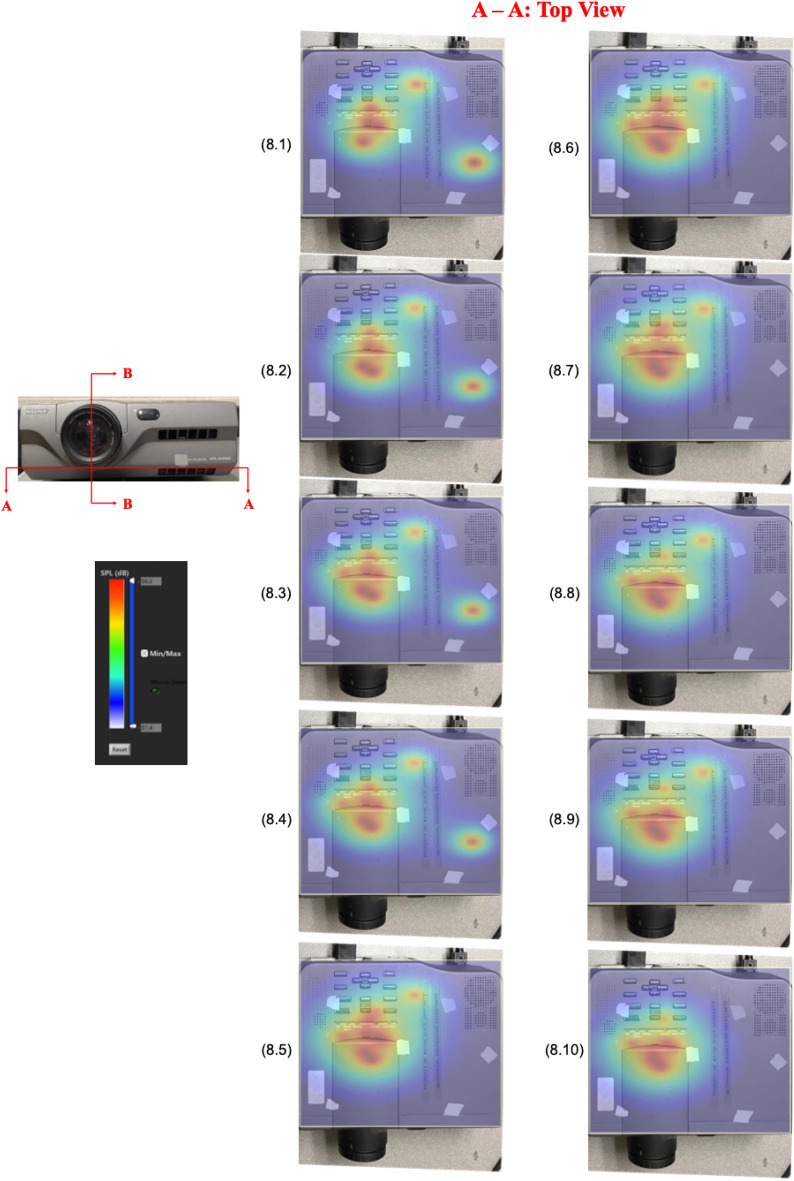
Evolution of the reconstructed acoustic pressure distributions on A-A cross section over the same time period, scanned at the same time interval, and the same color scale as that of B-B cross section. Results indicated that the acoustic energy stayed close to the blower blades, where fluid–structure interactions the strongest.

Figure 8 demonstrates the corresponding evolution of the aerodynamically-generated sound field on A-A cross section. Results indicated that the acoustic energies stayed close around the plane of blower blades, where fluid–structure interactions were the strongest.

It is emphasized that like Fig. 4, each frame of pictures in Figs. 7 and 8 depict not only sound source locations at specific time instances, but also distributions of the acoustic pressures generated by the blower. The amplitudes of the acoustic pressures are indicated by a color scale, red depicting the highest amplitude and blue the lowest amplitude of the acoustic pressure. Unlike the case as in Fig. 4, however, the source locations and acoustic pressure distributions changed with time. This is because in this case the sound field was generated aerodynamically. As a result, source locations and their strengths were not constant, but rather changing over time as airflow migrated toward the downstream direction.

## Discussion

Experimental validation results have shown that by using the laser-assisted see-through technology, we can locate sound sources and see sound field inside a solid structure, for which the line of sight is completely blocked and there are impedance discontinuities in the elastic media. The reason that this barrier in source localization can be broken is because the normal surface velocity is measured on an impenetrable surface, which is continuous across the thickness of the surface. In other words, the normal surface velocity on the front side is equal to that on the back side of the surface. Hence, we may take the input data measured on the front side and use them to locate sound sources behind this impenetrable surface. In this way, both sound sources and receivers are directly facing each other, as if the line of sight is unblocked and there are no impedance discontinuities in the elastic medium. Such a treatment is appropriate, especially when the interior space of a structure is small, so the direct sound waves are dominant. Once the source locations are specified, the interior acoustic pressure field can be reconstructed by using the HELS formulations, Eqs. (4), (5), (6), (7), (8), (9) and (10).

The discovery described above is unprecedented, whose importance cannot be overstated as it opens the door to a class of applications that are presently unattainable, for example, determining the root causes of noise and vibrations issues inside complex machines and structures. This is what we plan to do next. Also, we will apply this see-through technology to identify partial discharges that often occur inside high-voltage power transformer or electric equipment, which is a serious issue for the electric power industry. Currently, there are no effective ways to determine the precise location of partial discharges because they occur inside a high-voltage power transformer box.

With this see-through technology, we may have an effective solution to this and many other non-contact, non-invasive diagnostic and product quality control problems that cannot be accomplished easily by using traditional methodologies.

## Methods

### Hardware

The major pieces of hardware utilized in this study involved off-the-shelf items that included but not limited to: (1) six laser diodes Model LTD505T^70^; (2) six laser diode drivers; (3) six optical lenses Model F-C5-F3-1310^71^ for six laser diodes; (4) six photodiodes Model OP913SL^72^; (5) six photodiode transimpedance amplifiers Model LM-741^73^; (6) six beam splitters^74^ and (7) two NI (National Instrument) four-channel dynamic signal acquisition modules, Model NI9234^75^.

Note that the laser diode Model LTD505T was a low cost, single mode laser diode having a 5mW output power, 650 nm wavelength visible red beam, and 8 deg beam divergence after alignment. It required the measurement distance to be no more than 25 cm and retroreflective tapes to boost signal to noise ratios of the measured data. A more powerful laser diode may be utilized to produce desired input data at much farther distances without the help of retroreflective tapes.

In this study, the Cartesian coordinates of measurement points were specified manually. To improve the efficiency in data acquisition, a 3D scanner may be employed to acquire Cartesian coordinates of measurement points, for example, the off-the-shelf 3D scanners such as Polycam^76^, Lynx 3D Scanner^77^ and 3DMakerpro^78^.

### Software

The digitized normal surface velocity data along with measurement coordinates were taken as input to passive SODAR algorithms^79^, which was capable of locating multiple sound sources in 3D space simultaneously over the frequency range of 20–20,000 Hz. The algorithms can provide high spatial resolution in sound source localization (< 0.01 m @30 dB SNR over 20–20,000 Hz) within its effective working range.

The source locations together with the normal surface velocities were used to reconstruct the sound field inside a structure by using the HELS formulations^80^, which enabled one to acquire the acoustic quantities that include the acoustic pressures in 3D space and on the surface, the normal component of the time-averaged acoustic intensity, and the time-averaged acoustic power.

## Data Availability

The datasets generated and analyzed during this study are available in the Acoustics, Vibration, and Noise Control Laboratory repository at Wayne State University. They are available from the corresponding author on reasonable request.
